# Supplementary material for: Learning curve and surgical outcome of robotic assisted colorectal surgery with ERAS program
Source: Sci Rep. 2022 Nov 29;12:20566. doi: 10.1038/s41598-022-24665-w (PMC9709162; doi:10.1038/s41598-022-24665-w)
Supplement: Supplementary file 1 — Supplementary Information 1. [file 41598_2022_24665_MOESM1_ESM.zip › LSC Raw data-Ñ[▒K/070/P_ñw╝╨░O▒Kñσ.pdf]

[redacted]

Pathologic diagnosis:
1. Rectum, laparoscopic low anterior resection --- a. Status post polypectomy without residual tumor. The bilateral cut ends and perirectal lymph nodes (0/23) are free of tumor. b. Stitch granuloma and xanthomatous inflammation. c. Hyperplastic polyps.
2. IMA root, dissection --- Unremarkable (0/6)
3. Rectum, cut end, distal, resection --- Unremarkable.

Prognostic and predictive factor:
(Combined previous polypectomy: S0902049)
1. Tumor size: 1.6 cm. (in S0902049)
2. Depth of invasion: Submucosa. (in S0902049)
3. Histologic grade: G2: Moderately differentiated. (in S0902049)
4. Angiolymphatic invasion: Not identified.
5. Perineural invasion: Not identified.
6. Tumor deposits (discontinuous extramural extension): Not identified.
7. Macroscopic tumor perforation: Not identified.
8. Surgical Margins: a) Proximal Margin: Uninvolved by invasive carcinoma, distance of tumor from margin: 10.1 cm. b) Distal Margin: Uninvolved by invasive carcinoma, distance of tumor from margin: 2 cm. c) Radial or Mesenteric Margin: Uninvolved by invasive carcinoma, distance of tumor from margin: 1 cm.
9. Lymph node status: Uninvolved (0/29).
10. Extranodal involvement: Not applicable.
11. Tumor regression grading S/P CCRT: No known presurgical therapy.
12. Type of polyp in which invasive carcinoma arose: Tubulovillous adenoma. (in S0902049)
13. Additional pathologic findings: Not identified.
14. Pathological TNM Stage: pT1N0(According to the eighth edition, American Joint Committee on Cancer Staging Guidelines for Tumors).
15. TNM descriptors: Not applicable.

Gross description:
The specimen consists of 1) a segment of colon, 20 cm in length and 4.5 cm in proximal cut end circumference, 5 cm in distal circumference, and perirectal soft tissue. A post-polypectomy scar, 3cm in length, with granulation tissue, 0.7x0.3 cm, is noted, 2 cm to the distal cut end. On cross section, it is 10 mm to the radial margin. There are multiple tiny polyps, up to 0.3 cm, noted on mucosa. The both cut ends are free of tumor grossly. 2) IMA root, a piece of tan yellow soft tissue, 2.1x1.9x0.8 cm. 3) distal cut end, a piece of tan mucosa-covered soft tissue, 2.5x1.3x0.3 cm.
Representative part for section: A) proximal cut end B) distal cut end C-E) scar and granulation tissue F) polyps G) all of specimen 2 H) specimen 3.
#T-59600_2 #M-09183_2 0 1105 000000
#T-C4560_2 #M-09183_2 0 1105 000000

已電子簽章

敬告：本單僅供醫師參考使用，不具任何申請效力！！
請病患妥為保管，若不慎遺失需自負個人資料外洩責任！
